# Family Resources and Effects on Child Behavior Problem Interventions: A Cumulative Risk Approach

**DOI:** 10.1007/s10826-017-0777-6

**Published:** 2017-05-18

**Authors:** Truls Tømmerås, John Kjøbli

**Affiliations:** 1 Norwegian Center for Child Behavioral Development, P.O. Box 7053, Majorstuen, 0306 Oslo Norway; 2 Regional Center for Child and Adolescent Mental Health, Eastern and Southern Norway, P.O. Box 4623, Nydalen, 0405 Oslo Norway

**Keywords:** Family resources, Social risk, Cumulative risk, Behavior problems, Health care inequality, Evidence-based parent training interventions

## Abstract

Family resources have been associated with health care inequality in general and with social gradients in treatment outcomes for children with behavior problems. However, there is limited evidence concerning cumulative risk—the accumulation of social and economic disadvantages in a family—and whether cumulative risk moderates the outcomes of evidence-based parent training interventions. We used data from two randomized controlled trials evaluating high-intensity (*n* = 137) and low-intensity (*n* = 216) versions of Parent Management Training—Oregon (PMTO) with a 50:50 allocation between participants receiving PMTO interventions or regular care. A nine-item family cumulative risk index tapping socioeconomic resources and parental health was constructed to assess the family’s exposure to risk. Autoregressive structured equation models (SEM) were run to investigate whether cumulative risk moderated child behaviors at post-treatment and follow-up (6 months). Our results showed opposite social gradients for the treatment conditions: the children exposed to cumulative risk in a pooled sample of both PMTO groups displayed lower levels of behavior problems, whereas children with identical risk exposures who received regular care experienced more problems. Furthermore, our results indicated that the social gradients differed between PMTO interventions: children exposed to cumulative risk in the low-intensity (five sessions) Brief Parent Training fared equally well as their high-resource counterparts, whereas children exposed to cumulative risk in the high-intensity PMTO (12 sessions) experienced vastly better treatment effects. Providing evidence-based parent training seem to be an effective way to counteract health care inequality, and the more intensive PMTO treatment seemed to be a particularly effective way to help families with cumulative risk.

## Introduction

It is well established that behavioral problems in childhood (i.e., conduct problems, oppositional behaviors, and inattentive problems) negatively impact children’s long-term well-being through an association with school problems, work problems, social exclusion, and poor health (Maughan et al. 1985; Rutter et al. 1970; Sroufe et al. 2009). As with many other mental health-related problems, a social gradient has been established for behavior problems. Specifically, a family’s lack of social and economic resources has been found to be a social risk factor for the development and prevalence of such problems (Bøe et al. 2012; Mazza et al. 2016; Piotrowska et al. 2015; Sameroff et al. 1998). Furthermore, it has been recognized that there is a social gradient in the outcomes of mental health care services in general (i.e. health care inequality) and in the parent training interventions targeting behavior problems (Leijten et al. 2013; Lundahl et al. 2006; Pescosolido et al. 2013). Knowing that behavioral problems are one of the most frequent reasons for referrals to mental health services (Storvoll 1997; World Health Organization 2003), health care inequality in the services provided to children with behavior problems implies that we will fail to help high-risk children and that the effectiveness of these services will be reduced.

Health inequality remains a major societal challenge, and extensive research has examined how health care systems exacerbate these disparities (Spencer and Grace 2016). Service use, patient adherence, and service outcomes have been acknowledged as elements that are important for understanding health care inequality (Alegría et al. 2011), and lack of family resources has been an important focus of health care inequality research (Muntaner et al. 2013). In caring for behavior problems, social gradient approaches have commonly focused on family resources in the form of socioeconomic status (SES), typically assessed in terms of parental income and education level (Leijten et al. 2013). SES has been proposed as a “fundamental cause” of health inequality, structuring (un-) favorable mechanisms across contexts and diseases (Link and Phelan 1995; Muntaner et al. 2013). This implies that high SES families enjoy a vast number of flexible assets that they can use to their advantage to implement protective strategies and produce favorable treatment outcomes.

A more finely graded family resource approach may involve measurement of a wide array of social and economic resources, including parental mental and somatic health, that are associated with SES, (i.e., cumulative risk). Cumulative risk denotes a situation in which several social risk factors operate together. To give an example, comprehending a risk factor often involves envisioning the circumstances that accompany it. For instance, is having a single mother a risk if she has good health, a good income, and only one child? Probably not. However, if the single mother is poor, undereducated, and has three children, the picture is different. This example supports the fact that human beings often contend with constellations of risk factors rather than isolated instances of adverse circumstances (Seifer et al. 1992). In parent training, parents are the agents of change in their children (Forgatch and Patterson 2010). Thus, it is likely that limitations in parents’ access to resources create strain and stress on family life which in turn may create health care inequality.

Social gradients and thus health care inequality have been identified in interventions for behavior problems (Lundahl et al. 2006). In a meta-analysis, Reyno and McGrath (2006) found that single parent status, family size, low income, low education, and parental mental health issues diminished the effects of parent training. In another meta-analysis, Lundahl et al. (2006) found that low SES families benefitted less from parent training, particularly when the mode of treatment was group therapy. However, findings regarding family resources and the outcomes of parent training are inconsistent (Deković et al. 2011). For instance, low educational level, low marital satisfaction, maternal depression, and a lack of psychological resources have been found to enhance the benefits of treatment (Berlin et al. 1998; Gardner et al. 2009; Lundahl et al. 2006).

These conflicting findings regarding separate family risks have been taken as evidence that it is not the quality but the quantity of resources that is relevant to family functioning and children’s behavior (Rutter 2000; Sameroff et al. 1993; see Stolk et al. 2008, p. 57). However, we know of only two previous studies that have investigated how cumulative risk influences the effects of parent training interventions for addressing child behavior problems. In their study of a program aimed at reducing behavioral problems among children aged 1 to 3 years, Stolk et al. (2008) found no associations between cumulative risk and treatment effects. In studying another parenting intervention intended to promote cognitive development among low-birthweight infants, Liaw and Brooks-Gunn (1994) found that cumulative risk did not moderate treatment effects on behavior problems.

The social gradients in parent training outcomes are likely grounded in rings of social influence ranging from societal macro-level factors to micro-level factors such as individual characteristics and client-practitioner interactions (Spencer and Grace 2016). Hence, family resources and cumulative risk are proxies for different change mechanisms that operate at different levels of health care inequality and account for more complex situational decisions, rationalizations, and reasons for actions following parent training intervention. Given the findings of the above review, there is no consensus on whether family resources moderate the benefits of parent training interventions. Several change mechanisms are likely to vary according to family resources and thus impact treatment outcomes for behavior problems.

Following intervention, social network is one group of mechanisms that likely affects social gradients in treatment outcomes. According to Thoits (2011), socially graded network mechanisms may affect parents’ coping strategies. Low-resource families have less access to secondary networks of significant others who can offer various types of beneficial support, such as information and advice on interventions, encouragement, social influence, and role modeling based on past experiences (Smith and Christakis 2008; Thoits 2011). Sociocultural mechanisms may be another group of mechanisms that can reduce intervention benefits in socially graded patterns. Low-resource parents may hold more negative attitudes toward treatments and professional advice. Their norms, practices, and values may adhere less strictly to the parenting practices recommended in parent training (Gillies 2006), as parenting interventions may more closely match the resources and realities of middle-class households (Zilberstein 2016). Skill acquisition in parent training may also be less suited to low-resource parents’ modes of learning and loci of control (Pescosolido et al. 2013; Zilberstein 2016). Moreover, low-resource parents have been found to behave in a less self-assertive way when interacting with professionals, leading to differential outcomes that may disfavor their children (Gengler 2014; Weininger and Lareau 2003). Finally, different practical mechanisms, in the form of social and economic stressors (Conger et al. 1992), may reduce low-resource families’ potential to gain from interventions. Parents with cumulative risk are less likely to live in traditional two-parent families, and they might have poorer health and less money to pay for transportation expenses and child care, all of which could limit their access to the practical and social resources needed to participate in intervention and integrate the parenting strategies learned into their daily lives.

Although there is a general consensus that parent training interventions work better for high-resource families, some scholars have found compensatory effects (i.e., more positive effects) of parent training for the low-resource families (Leijten et al. 2013). Thus, several mechanisms are likely to be involved in beneficial compensatory patterns. Initial problem severity is a factor that could impact the benefits of treatment (Leijten et al. 2013) in that more severely troubled children may have more room for improvement. Similarly, the severity of a child’s problem might impact parental motivation and readiness to change (Baydar et al. 2003; DiClemente and Velasquez 2002). Features of the interventions could create compensatory effects for children from families with cumulative risk. Evidence-based interventions are based on a curriculum and follow a structured progression. Thus, there might be less room for high-resource parents to influence the treatment situation in an evidence-based intervention, where the teaching of core components is somewhat fixed. In that regard, evidence-based interventions might promote equality of care by ensuring that effective practices are provided to both rich and poor families (Cochrane 2004; Kristiansen and Mooney 2004). Moreover, the parent training interventions in focus are based on the Structured Interaction Learning model (SIL) of behavioral change (Forgatch and Patterson 2010). According to the SIL, family resources, and thus cumulative risk, affect the development of behavior problems by disrupting parenting style. Hence, children exposed to cumulative risk may have behavior problems that are more strongly induced by social risk and disrupted parenting. This implies that the SIL-based PMTO interventions might work better for families with cumulative risk, as their children’s behavior problem etiology might be more influenced by social risk environments and thus more in line with the PMTO curriculum. Relatedly, low-resource parents have more often been found to adhere to negative parenting styles (Elstad and Stefansen 2014). Hence, the parenting focus in interventions may be better suited to low-resource parents at the pre-intervention stage if they, to a greater degree than high-resource parents, lack parenting skills and abilities. If so, the practical focus on and rehearsal of parenting practices, particularly in the high-intensive intervention (explained in more detail below), could add a compensatory mechanism to the treatment experience of cumulative risk families.

In this study, we examined two evidence-based interventions differing in intensity (i.e., dosage and scope): the high-intensity PMTO Parent Group (hereafter called PMTO) and the low-intensity PMTO short form Brief Parent Training (BPT; when discussed together hereafter, these are called PMTO interventions). We focused on exposure to environments that were characterized by a lack of family social and economic resources in which we assessed the quantity of family resources, i.e., cumulative risk. The primary question raised was whether cumulative risk moderated the treatment effects of the PMTO interventions. Thus we elaborated on the conditions under which quantitative aspects of family resources exacerbate or ameliorate health care inequalities in (1) parent training vs. regular care and (2) a two-case comparison of the low-intensity BPT intervention and the high-intensity PMTO intervention.

## Method

### Participants

We used data from two randomized experiments evaluating PMTO and BPT interventions. They were designed as pretest (T1), posttest (T2; 8 weeks and 12 weeks after pretest for BPT and PMTO, respectively) and follow-up (T3; 6 months after post-test) parallel-group randomized trials with a 50:50 allocation ratio for the intervention and comparison groups. This implies that children and their parents were randomized to either one of the PMTO intervention groups or to the comparison groups receiving the regular care offered in the Norwegian health care system for children with behavior problems. The participants were randomized after completing the pretest questionnaire. Data collection occurred from 2007 to 2008 for BPT and from 2008 to 2009 for PMTO. Importantly, the recruitment and data-collection procedures were similar for both the BPT and PMTO groups. The participating children and families came from all five Norwegian health regions. The families had contacted the services themselves or had been referred by a primary care agency (e.g., child health clinics, child welfare agencies, schools or kindergartens). To mirror the regular referral procedures used in Norwegian health care services at that time, no formal screenings were used in this study. Thus, the inclusion of children and their families was based on practitioners’ clinical opinions after they had consulted with eligible parents. The participants were the parents (or caretakers) of children between the ages of 3 and 12 years.

Participant characteristics in the PMTO and BPT samples have been examined in a study by Tømmeraas 2016. Both samples overall contained participants that had lower economic and social resources compared to the Norwegian population of families with children. Regarding baseline differences between the two samples, the participant characteristics differed between the BPT and the PMTO sample. The descriptive statistics and baseline differences between the samples are described in Table 1. As Table 1 shows, the PMTO participants were clearly different from the BPT sample as they had more family risks and higher levels of behavior problems. Finally, we examined baseline differences related to treatment conditions in the two samples in terms of demographic and child behavior differences. In the PMTO sample there were no significant baseline differences between those receiving PMTO or the alternative of regular care. In the BPT sample, one difference emerged. Parent in the regular care group had on average higher education levels compared to the BPT group, *t*(185) = 2.47, *p* = 0.1.

**Table 1:** Descriptive statistics (means, standard deviations) and baseline group differences (chi-square and *t*-tests)

	PMTO	BPT	*t*	*p*	*Contrasts*
	*M* (SD)	*M* (SD)			
*Demographics*					
Household income^a^	63,7 (43.5)	67,4 (41.0)	0.6	0.543	ns.
Parent education^b^	2.2 (0.7)	2.4 (0.8)	3.1	0.002**	PMTO < BPT
Parent age	37.4 (6.3)	35.3 (6.1)	3.1	0.002**	PMTO > BPT
Cumulative risk	2.1 (1.6)	1.7 (1.8)	2.2	0.030**	PMTO > BPT
*Child characteristics*					
ECBI^c^	124.9 (27.9)	134.9 (31.2)	4.1	0.000***	PMTO > BPT
Child age	8.6 (2.4)	7.3 (2.3)	4.7	0.000***	PMTO > BPT
*Dichotomized demographics*	Percent (*%*)	Percent (*%*)			*Chi-square*
Single parents	32.8%	31.9%			ns.
Non-western^b^	8%	6%			ns.
Child gender	64% (boys)	68% (boys)			ns.
*N*	137	216			

^a^ Household income in USD divided by 1000. ^b^ Non-Western immigrant

^b^ Parent education level scale ranging from 1 (elementary school) to 4 (higher university degree)

^c^ Eyberg Child Behavior Inventory—intensity scale (ECBI) 36 item version (raw scores)

***p < 0.001; **p < 0.01; *p < 0.05

### Procedure

Both BPT and PMTO are part of a comprehensive evidence-based intervention program called TIBIR (Norwegian acronym; Early Initiatives for Children at Risk), which was developed to prevent and treat behavior problems in children (Solholm et al. 2013). PMTO targets children at moderate to high risk and is an intensive intervention consisting of 12 weekly sessions of 2.5 h each. The reduction of negative parenting and teaching and the rehearsal of positive parenting skills are central to PMTO; parents practice their parenting skills through role-play and participate in discussions. PMTO focuses on the following parenting skills: positive involvement, skill encouragement, family problem solving, monitoring, and effective discipline, which includes mild contingent sanctioning through ignoring and time-outs (or cool-downs). Moreover, much emphasis is placed on parent emotional control to reduce coercive interaction cycles between parents and children.

BPT is a low-intensity intervention that targets children between low and moderate risk and consists of up to five 1-h sessions. In these sessions, parents are taught only the most exigent parenting practices, much less time is devoted to skill rehearsal, and positive involvement and effective discipline are emphasized. Important differences between the two interventions are difference in dosage and comprehensiveness and the fact that PMTO is a group therapy. Both BPT and PMTO group and individual therapies have been tested in randomized effectiveness trials and have been shown to be more effective for reducing behavior problems than the practices regularly used in the Norwegian health care system for children with behavior problems (Kjøbli et al. 2013; Kjøbli and Ogden 2012; Ogden and Hagen 2008).

Regular care consisted of the following approaches: 63 families (35%) received no treatment, 25 families received help from school-based psychological services, 21 families received counseling from public health nurses, 22 families received counseling from public social workers in Norway’s welfare services, 2 families received behavioral counseling, 2 families received Marte Meo (Aarts 2000), and 4 families received other treatments (Kjøbli et al. 2013; Kjøbli and Ogden 2012). Overall, the regular care given to the comparison group varied in its content and intensity, and none of the participants in this group received other evidence-based treatments.

### Measures

Children’s externalizing behavior problems were measured with a 22-item version of the Eyberg Child Behavior Intensity scale (ECBI). We used this abbreviated version of the ECBI because previous studies have indicated that brief versions of the ECBI have better psychometric properties than the original scale (Hukkelberg et al. 2016). We created a child behavior problem latent construct, ECBI, based on three parcels of sum scores taken from the ECBI scale: inattentive behavior, 4 items (“Easily gets distracted”, “Has problems with concentration”); oppositional behavior, 10 items (“Does not follow rules without threat of punishment”, “Argues about rules”); and conduct problems, 8 items (“argues with similarly aged friends”, “destroys things”). Because of sample power issues and to maintain accurate identification, we used parcels in our measurement model, which is considered a better alternative than using the observed sum scores as outcomes (Rhemtulla 2016).

To measure the quantity of exposure to family risk, we constructed a cumulative risk index combining nine different family social and economic resources with parental health, as shown in Table 2. These resource indicators have been previously acknowledged as risk factors for the development of behavior problems and have been shown to affect health care outcomes (Alegría et al. 2011; Kjeldsen et al. 2014; Moffitt and Scott 2009; Narayanan and Nærde 2016; Piotrowska et al. 2015; Waldfogel et al. 2010; Zilberstein 2016). Our cumulative risk index had indicators similar to those used in the Sameroff cumulative risk index (Sameroff et al. 1987). Cumulative risk indexes are usually calculated by summing the number of dichotomized risk factors (Evans et al. 2013; Trentacosta et al. 2008). We computed our cumulative risk index using the nine dichotomized indicators shown in Table 2. Parents scored an indicator as “1” if it was present and “0” if it was absent. Cut-offs were based on previously established limits and/or corresponded to previous cumulative risk research or population-validated numbers (for mental health; Evans et al. 2013; Reedtz et al. 2008; Trentacosta et al. 2008). The cumulative risk indicator was based on parent-reported information assessed before treatment.

**Table 2:** Cumulative risk indicators, definitions, and percentages complying with sample criteria

Indicator:	Description of criteria	%
OECD poor^a^	OECD 50% of median income	25.8
Low education	Did not finish upper secondary school	23.5
Unemployed	Financial unemployment support	6.2
Non-Western immigrant	From Eastern Europe or south of the equator	6.8
Single parent	One caregiver in the family	32.3
Young caregiver	Parent ≤ 21 years of age	10.2
Caregiver ratio	Ratio ≤ 0.5 adults per child	28.3
Somatic health^b^	Cut off at ≥3	18.1
Mental health	Average score cut off ≥ 2	35.4

^a^ Organization for Economic Co-operation and Development (OECD) equivalence poverty scale

^b^ One-item scale ranging from 1 (excellent health status) to 5 (poor health status)

^c^ Adjusted SCL-5 scores ranging between 1 and 5, a higher score indicates more anxiety and mental distress

The poverty measure, OECD poor, was based on the OECD equivalence scale (Organisation for Economic Co-operation and Development 2016); families with less than 50% of the median net income were coded as poor. Thus, the different poor cut-offs were calculated as a function of family constellation and the 2008 average population median net income of approximately 27,500 USD. The low education variable was a dichotomized variable derived from a categorical education level variable counting; 1 = elementary school; 2 = upper secondary school; 3 lower university degree; and 4 higher university degree (>4 years). Parents that scored 1 on the education level variable were coded as 1 in the low education cumulative risk indicator. Parents’ mental health was measured with the Symptom Checklist 5 (SCL-5), which measures anxiety and depression. SCL-5 is a (very) short-form of the SCL-25 mental health index which is derived from the SCL-90 psychopathology rating scale (Derogatis 1992; Strand et al. 2003). In a Norwegian population-based sample, Strand et al. (2003) found that the correlation between SCL-5 and SCL-25 was 0.91. SCL-5 risk cut-offs used were based on numbers validated and normed in a Norwegian study (Tambs and Moum 1993). Cronbach’s alpha for the SCL-5 was 0.88. The variable measuring parents’ somatic status was a one-item and non-validated scale ranging from 1 (“Excellent health status”) to 5 (“Very poor health status”). Table 3 displays the bivariate correlations among the cumulative risk indicators.

**Table 3:** Bivariate correlations between family factors and child behavior problems

Variable:	1	2	3	4	5	6	7	8	9
1. OECD poor^a^	1								
2. Low education	0.25***	1							
3. Unemployed	0.22***	0.13*	1						
4. Non-Western	0.15**	−0.04	0.07	1					
5. Single parent	0.44***	0.15**	0.17***	0.05	1				
6. Caregiver ratio	0.14**	0.26***	−0.01	0.06	0.11*	1			
7. Young parent	0.41***	0.17***	0.18***	0.01	0.60***	0.02	1		
8. Somatic health	0.16**	0.18***	0.03	0.14*	0.13*	0.01	0.05	1	
9. Mental health	0.20***	0.13*	0.10	0.11*	0.07	0.04	0.09	0.34***	1

^a^ Organization for economic Co-operation and development (OECD) equivalence poverty scale

Risk seemed to cluster in our sample. Hence, being poor was significantly correlated with all the other nine risk factors, as shown in Table 3. Moreover, the cumulative risk index was significantly correlated with higher baseline levels of behavior problems (*r* = 0.17, *p* = 0.002), meaning that children who were exposed to cumulative risk in their families had, on average, higher levels of behavior problems before treatment.

### Data Analyses

**Fig. 1:**
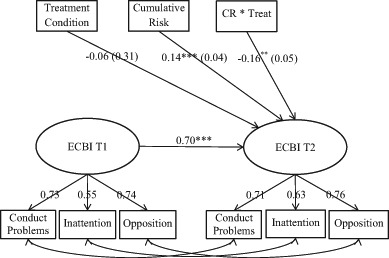
Cumulative risk and child behavior change in PMTO interventions vs. regular care. Autoregressive SEM analysis, posttreatment (T2) regressed on pretreatment behavior (T1). *Note:* Eyberg Child Behavior Inventory—intensity scale (ECBI). Interaction variable Cumulative Risk multiplied by Treatment Condition (CR * Treat). Coefficients were standardized on *Y* (equals Cohen’s *d*), *standard error* is displayed in parentheses. Model fit information: *X* ^2^(df)* = *27.0 (20), CFI = 0.99 TLI = 0.99 RMSEA = 0.06. ***p < 0.001; **p < 0.01; *p < 0.05

**Fig. 2:**
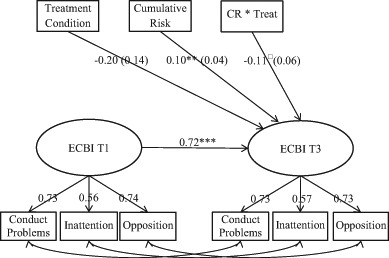
Cumulative risk and child behavior change in PMTO interventions vs. regular care. Autoregressive SEM analysis, follow-up (T3) regressed on pretreatment behavior (T1). *Note:* Eyberg Child Behavior Inventory—intensity scale (ECBI). Interaction variable Cumulative Risk multiplied by Treatment Condition (CR * Treat). Coefficients were standardized on *Y* (equals Cohen’s *d*); standard errors are displayed in parentheses. Model fit information: *X* ^2*(*^df)* = *36.3 (20), CFI = 0.98 TLI = 0.97 RMSEA = 0.05. ***p < 0.001; ** *p* < 0.01; *p < 0.05; ^†^ = 0.059

Children were the unit of analysis in this study. Social gradients in the outcomes of PMTO, namely, whether cumulative risk moderates treatment effects, were examined with autoregressive SEM analysis using Mplus 7 (Muthén and Muthén 2012). We ran the models as intent-to-treat analyses to examine intervention effects across treatment conditions using the three time-points: T1, T2, and T3. The outcomes in our models showed changes in the children’s behavior problems from baseline levels. The SEM models are displayed in Fig. 1 and Fig. 2. The treatment variable shows the treatment effects for families that scored 0 on the cumulative risk variable, the cumulative risk variable displays the effects of exposure to cumulative risk for the regular care group, and the interaction term cumulative risk * treatment shows the treatment effects for the families who were exposed to cumulative risk in the PMTO groups.

The measurement model, displayed in Figs. 1 and 2, shows the standardized factor loadings and correlated error terms for the three parcels constituting our latent outcome variable (ECBI). The error terms in the three parcels were correlated across the time points. These unanalyzed associations represent shared sources of variability over and above the latent factors. To investigate whether there were any sample-specific differences due to differences in cumulative risk and the intensity of treatment (i.e., dosage and comprehensiveness), we ran auto-regressive multi-group SEM models analyzing the BPT and the PMTO interventions separately. We used several goodness-of-fit indexes to evaluate our theoretical model fit: chi-square statistics, the comparative fit index (CFI), the Tucker-Lewis index (TLI), and the root-mean-square error of approximation (RMSEA; see table and figure notes). Moreover, we checked our data for potential outlier observations through a visual inspection of residual plots and the estimation of a five percent trimmed mean in the outcome variable. Neither of these procedures indicated that the effects of outliers biased our results.

Additionally, we performed several sensitivity tests to inspect functional forms in our data and to address potential rival conclusions. We also included child age and gender as covariates in our analyses and tested non-linear patterns in our data. Moreover, we evaluated the family risk factors in the cumulative risk index using independent-additive models to investigate the unique effects of each cumulative risk indicator. Finally, we partialled out the control group families that received no treatment to determine whether the cumulative risk comparison group estimates were influenced by receiving active treatment or no treatment.

There was little attrition in our sample. Of the 353 participating families, 301 (85%) completed T2, and 275 (78%) completed T3. When comparing the attrition group with the completers in each trial, few differences in intake characteristics emerged (for more details see Kjøbli et al. 2013; Kjøbli and Ogden 2012). A missing data analysis, Little’s MCAR test, indicated that the missing data were missing completely at random. Thus, we modeled the data using full-information maximum likelihood, which uses all the available information from the observed data to handle missing data (Wothke 2000).

## Results

**Fig. 3:**
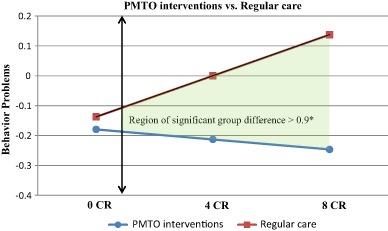
Simple slopes and region of significance for the interaction between treatment conditions and cumulative risk, child behavior change at T2

The post treatment effects (T2) for the pooled sample of PMTO interventions are displayed in Fig. 1. The results showed that the children in the PMTO group from families with one additional cumulative risk generally experienced more benefit from treatment; in T2, behavior problems were reduced by an average of 16% of a standard deviation for each accumulated risk (*ß* = −0.16, *p* < 0.01; results were standardized on *Y* only). Conversely, for the regular care group, scoring higher on cumulative risk was significantly associated with lower treatment benefits; this group displayed increased levels of problem behavior in T2 (*ß* = 0.14, *p* =  < 0.001). This implies that one additional cumulative risk entailed an increase of 14% of a standard deviation in children’s behavior problems in T2. In Fig. 2, the pooled PMTO group results were not significantly replicated at T3 (*ß* = −0.11, *p* = 0.06); however, the coefficient had a considerable size in a similar direction as in T2. For the regular care group, the T2 results were replicated at T3, (*ß* = 0.104, *p* < 0.01). Overall, the model fit was within an acceptable range for the fit indexes in all the estimated models (Hu and Bentler 1999); see table notes. The factor loadings from the parcels in all the SEM measurement models were >0.50; see Figs. 1 and 2. Moreover, for the Fig. 1 results, we computed the simple slopes and calculated the regions of significance; see Fig. 3 (Preacher et al. 2006). Differences between the groups were significant for cumulative risk scores above 0.9, meaning that group differences between those who received parent training and those who received regular care were significant for families with one or more risks.

**Fig. 4:**
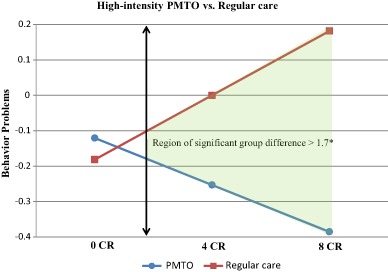
Simple slopes and region of significance for the interaction between treatment condition and cumulative risk for the high-intensity PMTO sample, child behavior change at T2

Next, we examined whether there were sample-specific differences in the associations between cumulative risk and changes in behavior problems and whether the treatment effects differed for the families with cumulative risks according to treatment intensity. The path coefficients revealed such differences; see Table 4. The PMTO intervention seemed to be particularly effective for children from families with cumulative risks at both T2 (*ß* = −0.33, *p* =  < 0.001) and T3 (*ß* = −0.30, *p* =  < 0.001). The low-intensity BPT intervention results did not reveal any significant changes in the treatment effects for the families with cumulative risk. In both samples, the children who received regular care experienced significant increases in behavior problems at all time points; see Table 4. We computed the region of significance for the path T1 → T2 in the PMTO sample. We found that group difference between the PMTO group and comparison group was significant for cumulative risk scores of 1.7 and higher (Fig. 4; Preacher et al. 2006).

**Table 4:** Autoregressive multi-group SEM analysis displaying separate path coefficients for the BPT and the PMTO samples

Parameter	BPT		PMTO	
	*ß*	SE	*ß*	SE
Model 1^a^: ECBI T2				
ECBI T1	0.69***	0.05	0.69***	0.07
Treatment	−0.22	0.16	0.26	0.24
Cumulative risk	0.12**	0.04	0.19**	0.07
Treat * CR	0.08^ns^	0.07	−0.33***	0.09
Model2^b^: ECBI T3				
ECBI T1	0.72***	0.05	0.68***	0.07
Treatment	−0.29	0.16	−0.03	0.25
Cumulative Risk	0.08	0.04	0.18*	0.08
Treat * CR	0.00	0.07	−0.30**	0.10

Eyberg Child Behavior Inventory—intensity scale (ECBI). Interaction variable Cumulative Risk multiplied by Treatment Condition (CR * Treat). ^a^ Model 1 ECBI T2 regressed on T1, model fit information: *X* ^2^(df)* = *55.9 (48), CFI = 0.99 TLI = 0.99 RMSEA = 0.05

^b^ Model 2 ECBI T3 regressed on T1, model fit information: *X* ^*2(*^df)* = *59.0 (48), CFI = 0.99 TLI = 0.98 RMSEA = 0.04

Coefficients were standardized on *Y* (equals Cohen’s *d*)

***p < 0.001; **p < 0.01; *p < 0.05

Additionally, we performed several sensitivity tests to gauge the robustness of our conclusions. First, we examined whether there were non-linear patterns in the cumulative risk associations with changes in behavior problems. We tested both a cubic parameterization of cumulative risk and threshold effects for families with between 2 and 5 cumulative risks. No threshold or non-linear patterns were significantly different from 0 (results available upon request). Moreover, we tested whether cumulative risk had unequal effects according to the child’s gender and age. Both variables were entered into the analysis as covariates and into a three-way interaction term with treatment and cumulative risk (results available upon request). No significant effects of gender or age emerged. Furthermore, we tested the nine cumulative risk factors singularly in independent-additive models to test for unique predictive validity. We found that no significant results emerged from these analyses. Finally, 35% of our control group cases received no treatment. We suspected that these children and families biased our estimates, and we ran additional analyses without these 63 families. The results were similar to those of the original models in terms of both the coefficient sizes and significance levels, and the full sample was thus included in our final analysis.

## Discussion

In this study, we extended the literature on health care inequality in behavior problem interventions by examining the relationships between family cumulative risk and treatment outcomes in evidence-based parent training and regular care. We also examined cumulative risk associations in a case comparison between low-intensity and high-intensity PMTO. First, we found that exposure to cumulative risk differentially moderated the treatment effects of PMTO interventions and regular care, as reflected by the opposite social gradients of the changes in the children’s behavior problems. The children who received PMTO interventions and were exposed to one or more cumulative risks experienced compensatory effects, meaning that the children from families with low amounts of resources experienced greater reductions in their behavior problems than the children from high-resource families. Conversely, the regular care group exposed to equal levels of risk experienced more behavior problems over time, indicating that the children from low-resource families had poorer treatment outcomes with regular care than the children from high-resource families. Second, we found that the families with cumulative risk benefitted differently according to the intensity of the PMTO treatments; the children who were exposed to cumulative risks experienced vast improvement with high-intensity PMTO. Thus, cumulative risk produced social gradients in treatment effects according to both the treatment conditions and the treatment intensity.

The effects of cumulative risk seem to be linear, and we conclude that it is the sheer number of risk factors that changes the treatment effects rather than differences in treatment effects below and above a certain threshold. Previous research regarding cumulative risk and the effects of parent training is both limited and inconsistent (Liaw and Brooks-Gunn 1994; Stolk et al. 2008). In this sample of at-risk children, our results revealed opposite social gradients, indicating that children from families with cumulative risk were highly receptive to the type of care provided by the Norwegian health care system.

The finding of opposite social gradients for the treatment effects, related to the PMTO interventions and regular care, implies that type of treatment may either create or reduce health care inequality among low-resource families receiving help for their child’s behavior problem. Hence, there seem to be different mechanisms related to changes in health care outcomes for those who receive parent training and those who receive regular care. Unfortunately, we have limited knowledge about the contents of regular care. However, we know that the differences between PMTO interventions and regular care are rooted in the differences between structured, curriculum-grounded, evidence-based parent training and the more unstructured parent counseling provided in regular care. Hence, the mechanisms related to inequality in regular mental health care and parent training interventions, such as network mechanisms, sociocultural mechanisms and practical mechanisms (Alegría et al. 2011; Spencer and Grace 2016; Zilberstein 2016), applied more to the regular care group in our study. It might be that these mechanisms come into play more when the mode of treatment is less structured and does not explicitly target parenting style. Moreover, this could indicate that there was more room for high-resource parents to influence treatment content—and thus their children’s outcomes—under the less structured health care conditions.

Conversely, the compensatory effects of PMTO interventions for low-resource families indicate that other beneficial mechanisms were operating within these structured treatment conditions. It might be that the children from low-resource families were more exposed to disrupted parenting practices and that systematic parent training was more adapted to their pre-intervention skills and family climate. Moreover, separate analysis of our preventive BPT and high-risk PMTO samples revealed that the compensatory effects were more prominent under the latter treatment condition. In the BPT sample, the families with both low and high cumulative risk experienced positive changes in their children, whereas in the more intensive PMTO treatment group, the families with high cumulative risk experienced a vast improvement. The reduction in their children’s behavior problem levels had the effect size of approximately 30% of a standard deviation change per level increase in cumulative risk, which underpins this argument. It seems that providing more intensive treatment to the more troubled families exaggerates the compensatory effect mechanisms. Thus, there is probably interplay between compensatory mechanisms, such as parent’s pre-intervention parenting skills, the etiology of child behavior problems, and readiness for change, that produces favorable outcomes for the families with cumulative risk in the PMTO treatment. However, more research is needed to reveal the mediational relationships behind these compensatory patterns.

Behavior problems in childhood contribute to social gradients in child well-being, but behavior problems are also partly the products of social disparities. Low-resource backgrounds have been found to increase the risk that a child will experience behavior problems (Piotrowska et al. 2015), and behavior problems themselves have negative long-term developmental impacts, as children are exposed to multiple threats to their well-being later in life (Moffitt et al. 2002). From a mental health care perspective, this underscores the need to provide effective care for this vulnerable group of children and their families. If we fail to do this, mental health care interventions aimed at behavior problems will certainly produce health care inequality and exacerbate existing health inequality (and ultimately social inequality) among low-resource populations. The opposing social gradients we found in care for behavior problems support this argument; the type of treatment provided can either produce or reduce health care inequality.

The steady negative development displayed by children from low-resource backgrounds in the regular care group is thus consistent with the theory of family resources as a “fundamental cause” structuring flexible assets when coping with children with behavior problems (Link and Phelan 1995; Muntaner et al. 2013). This negative development is also consistent with behavior problems risk theory, which postulates that social risk, in the form of family resources, intensifies the development of behavior problems (Mazza et al. 2016). When helping children at risk for such negative development, evidence-based parent training interventions seem to be an efficient strategy for counteracting health care inequality and the lack of family resources as a “fundamental cause” and thus for effectively preventing and altering negative developmental trajectories for children from low-resource backgrounds. It has been postulated that evidence-based treatments can promote equality in care (Cochrane 2004; Kristiansen and Mooney 2004). Regarding the outcomes of treatment, our results support this postulation.

### Limitations

In this study, we had the advantage of using experimental data gathered in the Norwegian regular care system for children with behavior problems. However, several limitations should be considered. Admittedly, combining risk factors into a cumulative risk index will, to some extent, obscure the etiology of social risk. We also applied an equal weights assumption when we pooled the dichotomized risk factors, and we combined risk factors from different domains, such as family demographics and parental health. However, we can offer insights into how different amounts of risk exposure affect health care outcomes for children with behavior problems. The predictive validity of our cumulative risk index supports this notion. Moreover, although we adhered to previously established cut-offs in cumulative risk research, one may still argue that the process of dichotomization inflicts arbitrary limits on risk factors. Nevertheless, evidence is very limited regarding cumulative risks and the outcomes of care for behavior problems. Thus, the effects of cumulative risk must be replicated in other samples and contexts. Moreover, the RCT design evaluating treatment intervention packages, such as PMTO, did not allow us to address which treatment components that produced the compensatory effects of PMTO (Collins 2014). Other approaches, such as factorial designs, might be more appropriate for elaborating further on the change mechanisms related to cumulative risk that is at work in parent training.
